# A novel PI3K inhibitor PIK-C98 displays potent preclinical activity against multiple myeloma

**DOI:** 10.18632/oncotarget.2688

**Published:** 2014-12-02

**Authors:** Jingyu Zhu, Man Wang, Yang Yu, Huixin Qi, Kunkun Han, Juan Tang, Zubin Zhang, Yuanying Zeng, Biyin Cao, Chunhua Qiao, Hongjian Zhang, Tingjun Hou, Xinliang Mao

**Affiliations:** ^1^ Jiangsu Key Laboratory of Translational Research and Therapy for Neuro-psycho-diseases, Department of Pharmacology, College of Pharmaceutical Sciences, Soochow University, Suzhou, China; ^2^ Jiangsu Key Laboratory of Preventive and Translational Medicine for Geriatric Diseases, Soochow University, Suzhou, China; ^3^ Department of Medicinal Chemistry, College of Pharmaceutical Sciences, Soochow University, Suzhou, China; ^4^ Department of Pharmaceutical Analysis, College of Pharmaceutical Sciences, Soochow University, Suzhou, China; ^5^ Department of Medicinal Chemistry, School of Pharmaceutical Sciences, Zhejiang University, Hangzhou, China

**Keywords:** phosphatidylinositol-3-kinase, multiple myeloma, C98, virtual screen

## Abstract

Recent clinical trials have demonstrated targeting PI3K pathway is a promising strategy for the treatment of blood cancers. To identify novel PI3K inhibitors, we performed a high throughput virtual screen and identified several novel small molecule compounds, including PIK-C98 (C98). The cell-free enzymatic studies showed that C98 inhibited all class I PI3Ks at nano- or low micromolar concentrations but had no effects on AKT or mTOR activity. Molecular docking analysis revealed that C98 interfered with the ATP-binding pockets of PI3Ks by forming H-bonds and arene-H interactions with specific amino acid residues. The cellular assays demonstrated that C98 specifically inhibited PI3K/AKT/mTOR signaling pathway, but had no effects on other kinases and proteins including IGF-1R, ERK, p38, c-Src, PTEN, and STAT3. Inhibition of PI3K by C98 led to myeloma cell apoptosis. Furthermore, oral administration of C98 delayed tumor growth in two independent human myeloma xenograft models in nude mice but did not show overt toxicity. Pharmacokinetic analyses showed that C98 was well penetrated into myeloma tumors. Therefore, through a high throughput virtual screen we identified a novel PI3K inhibitor that is orally active against multiple myeloma with great potential for further development.

## INTRODUCTION

Multiple myeloma (MM) is a kind of plasma cell malignancy that accounts for more than 10% of all hematologic cancers and 2% of cancer-related death [1]. The past decade has witnessed several novel drugs including the proteasome inhibitors (bortezomib and calfizomib) and the immunomodulators (thalidomide and its analogs) for MM treatment, however, the death rate remains high. American Cancer Society estimates that there are approximately 22,350 new cases of MM and 10,710 MM-related deaths in USA in 2013 [2]. Therefore, novel treatments are urgently demanded.

In recent years, substantial efforts have been made to identify optimal targets for MM treatment, and the phosphatidylinositol-3 kinase (PI3K)/AKT signaling pathway comes to attention. PI3Ks are a group of lipid kinases, of which Class I including p110α, p110β, p110δ, and p110γ is involved in carcinogenesis and chemoresistance of multiple cancers [3–6]. Dysregulated PI3K signaling pathway is a frequent event in MM, including (1) increased insulin-like growth factor 1 (IGF-1) and interleukin-6, both of which promote MM cell proliferation and survival [7, 8]; (2) silenced phosphatase and tensin homolog (PTEN) that antagonizes the PI3K/AKT signaling [9]; (3) overactivated PI3K/AKT [10]. Dysregulation of the PI3K/AKT signaling pathway contributes to MM pathophysiology, chemoresistance and poor prognosis, while interference with this pathway results in MM cell death [10].

Currently dozens of inhibitors of PI3K and AKT are under development and some have been successfully moved to clinical trials for the treatment of various hematological malignancies, including leukemia and MM [10, 11]. The phase II clinical trials showed that as a single agent, idelalisib, an inhibitor of PI3Kδ, reached a total 57% of response rate in relapsed indolent lymphoma and 72% of overall complete rate in relapsed chronic lymphocytic leukemia [12, 13]. These studies have significantly accelerated the marketing of idelalisib for the treatment of refractory leukemia and lymphoma which was approved by Food and Drug Administration of USA on July 23, 2014. Therefore, targeting PI3K is emerging as a high promise for the treatment of these diseases.

In the present study, we took advantage of the advancement of PI3K crystal structure, molecular docking, and combinatorial chemistry, and performed a high throughput virtual screen against 800,000 small molecule compounds using PI3Kγ as the subject and identified a promising PI3K inhibitor with a novel scaffold for MM treatment.

## RESULTS

### C98 potently inhibits PI3K activity

**Figure 1:**
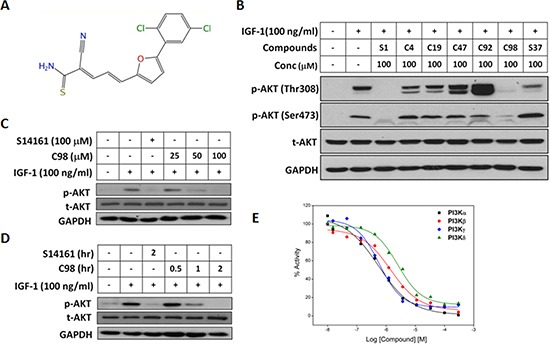
C98 inhibits PI3K activity **(A)** Chemical structure of C98. **(B)** OPM2 cells were starved overnight and then treated with six compounds identified from the virtual screening for 2 hr, followed by IGF-1 stimulation for 15 min. Cell lysates were prepared for AKT expression and phosphorylation analyses. The known PI3K inhibitor S14161 was used as a positive control. **(C)** OPM2 cells were starved overnight, then treated with C98 at indicated concentration (25, 50 and 100 μM) or S14161 (100 μM) for 2 hr, followed by IGF-1 (100 ng/mL) stimulation for 15 min Cell lysates were prepared for AKT expression and phosphorylation analyses. **(D)** OPM2 cells were starved overnight and then treated with C98 (100 μM) for the indicated time periods (0.5, 1, 2 hr) or S14161 (100 μM) for 2 hr, followed by treatment with 100 ng/mL of IGF-1 for 15 min Cell lysates were prepared for AKT expression and phosphorylation analyses. **(E)** PI3K activity analyses in a cell-free system. Increasing concentrations of C98 were incubated with the recombinant PI3K isoforms α, β, δ, and γ, respectively. Activity of each kinase was determined with HotSpot technology as described in the Methods section “Kinase activity in cell-free assay”.

Six novel compounds which were identified as potential PI3K inhibitors of which C96 has been demonstrated to induce MM cell death by targeting the PI3K signaling [16]. To determine whether C98 was able to inhibit PI3K activity, a MM cell line OPM2 which displays high PI3K activity due to lack of PTEN, a negative modulator of the PI3K signaling pathway [16], was starved overnight followed by C98 treatment and subsequent stimulation with IGF-1, a critical trigger of PI3K signaling and a key growth factor in MM cell proliferation. Immunoblotting analysis revealed that C98 potently inhibited PI3K activity. As shown in Figure 1B, C98 suppressed AKT phosphorylation in a manner similar to a proven PI3K inhibitor S14161 [15]. More importantly, C98 inhibited AKT phosphorylation in a time- and concentration-dependent manner in OPM2 cells (Figures 1C and 1D). To confirm this hypothesis, the inhibitory potency of C98 on PI3K enzymes was measured in a cell-free system with individual recombinant human PI3K isoforms [15]. The results revealed that C98 inhibited all Class I isoforms (Figure 1E). The IC_50_ values against α, β, δ, and γ isoforms were 0.59, 1.64, 3.65, and 0.74 μM, respectively. To further evaluate its specificity on PI3K, the effects of C98 on other associated kinases AKT, PDK1, and mTOR were also measured in the enzymatic systems. The results showed that C98 displayed minimal inhibitory effects on AKT activation (IC_50_ = 61.4 μM) but failed to suppress PDK1 and mTOR activity even at a concentration of 300 μM. Therefore, C98 preferred to inhibit PI3K activity.

### C98 specifically inhibits the PI3K signaling pathway

**Figure 2:**
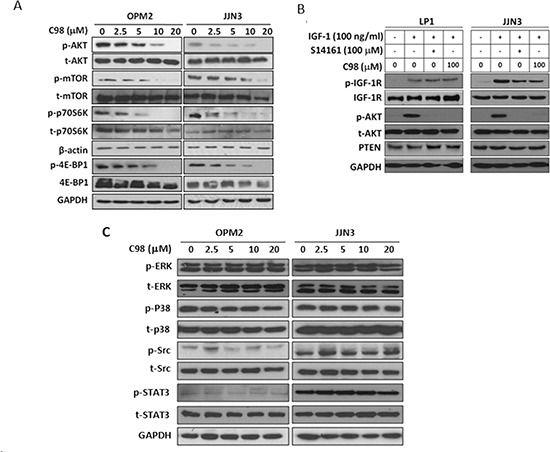
C98 specifically inhibits PI3K/AKT/mTOR pathway **(A)** OPM2 and JJN3 cells were treated with increasing concentration of C98 for 24 hr before subject to immunoblotting against p-AKT, t-AKT, p-mTOR, mTOR, p-P70S6K, P70S6K, p-4E-BP1, and 4E-BP1. **(B)** LP1 and JJN3 cells were starved overnight, then treated with C98 or S14161 for 2 hr, followed by IGF-1 (100 ng/mL) for 15 min. Cells were then prepared for the analysis of the expression of p-AKT, t-AKT, p-IGF-1R, and IGF-1R by immunoblotting. **(C)** OPM2 and JJN3 cells were treated with increasing concentrations of C98 for 24 hr before being applied to immunoblotting against p-ERK, t-ERK, p-P38, t-P38, p-Src, t-Src, p-STAT3, or t-STAT3. GAPDH and β-actin were used as loading controls.

Subsequently, the inhibition of C98 on the PI3K signaling pathway was evaluated in MM cell lines OPM2 and JJN3. Immunoblotting analyses revealed that C98 downregulated the phosphorylation levels of AKT, mTOR, p70S6K, and 4E-BP1 (Figure 2A). Because all these kinases are downstream signaling proteins in the PI3K pathway while C98 had no effects on AKT and mTOR in the cell free assay, the inhibition of C98 on these kinases was probably due to its suppression on PI3K.

To find out whether C98 also interfered with other signaling pathways, we first checked the receptor tyrosine kinase IGF-1R, which mediates PI3K activation in MM cells in the presence of IGF-1. Starved MM cells LP1 and JJN3 were incubated with C98 followed by IGF-1 stimulation. Immunoblotting assays revealed that IGF-1 induced phosphorylation of both IGF-1R and AKT (Figure 2B), and C98 suppressed AKT phosphorylation but had no effects on IGF-1R activation at a concentration of 100 μM (Figure 2B). This result suggested that C98 inhibited PI3K independent of IGF-1R activation. Moreover, we also measured the expression of PTEN, a negative regulator of PI3K signaling, in MM cells after C98 treatment. The immunoblotting analysis showed that C98 had no effects on the PTEN protein level (Figure 2B).

To further clarify the effects of C98 on other signaling pathways associated with PI3K signaling, we evaluated ERK and p38, both of which mediate extracellular signaling similar to PI3K. As shown in Figure 2C, C98 failed to decrease the phosphorylation levels of both ERK and p38. Moreover, C98 failed to inhibit the phosphorylation of c-Src, a non-receptor kinase (Figure 2C). We also checked the phosphorylation status of STAT3, a signal transducer and activator of transcription. C98 did not decrease phosphorylated STAT3 (Figure 2C). Therefore, C98 had no effects on these kinases/proteins associated with PI3K signaling. These results suggested that PI3K was the target of C98.

### Modeling of C98 interactions with PI3K isoforms

**Figure 3:**
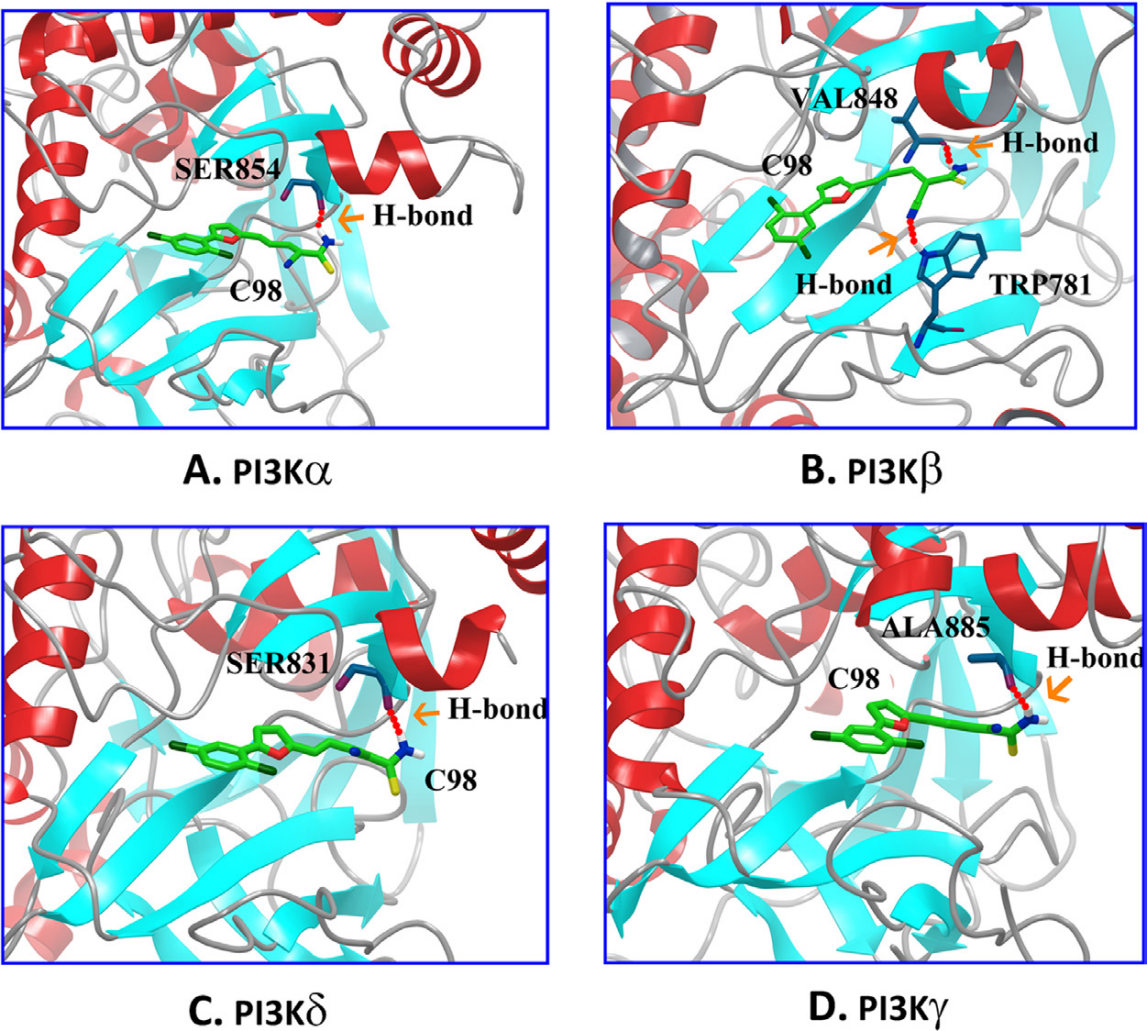
Molecular modeling of the C98/PI3Ks complexes 3-D presentations of the interactions between C98 and PI3Kα **(A)**, PI3Kβ **(B)**, PI3Kδ **(C)**, and PI3Kγ **(D)**. Residues of PI3Ks: blue, C98: green, and H-bond: red.

We next analyzed the interaction of C98 with PI3K isoforms by computer modeling. It showed that the binding geometries of C98 in the ATP pockets of PI3K isoforms were quite similar. For example, the amino group of C98 could form H-bonds with the specific residues of each isoform: Ser854 of α (Figure 3A), Val848 of β (Figure 3B), Ser831 of δ (Figure 3C), and Ala885 of γ (Figure 3D). In addition, the nitrile group of C98 as an H-bond acceptor could form an H-bond with Trp781 of PI3Kβ (Figure 3B). All these H-bonds played a key role in the interaction between C98 and PI3Ks because the H-bonds acted as a gate to prevent C98 from escaping from the binding sites [25].

To further recognize the dynamic interaction patterns between C98 and PI3Ks, MD simulations and free energy decomposition analyses were employed. As illustrated in Supplementary Figure S1, in addition to the H-bonds (Figure 3), the arene-H interactions were also formed between C98 and specific residues in each isoform, including Tyr836/Ile848/Val850/Ile932 of PI3Kα (Supplementary Figure S1A), and Ile930 of PI3Kβ (Supplementary Figure S1B), Tyr813/Ile910 of PI3Kδ (Supplementary Figure S1C), and Tyr867 of PI3Kγ (Supplementary Figure S1D). Moreover, the *p*-chlorophenylmoiety and the furan ring of C98 also formed van der Waals interactions with PI3K isoforms (Supplementary Figure S2). All these lipophilic interactions between the benzene and furan rings and the cavity lined by hydrophobic residues Ile848/Ile932 of PI3Kα, Ile845/Ile930 of PI3Kβ, Ile825/Ile910 of PI3Kδ, or Ile879/Ile963 of PI3Kγ were important to stabilize the interaction between C98 and PI3K isoforms [26].

### C98 induces apoptosis of MM cells in associationwith its PI3K inhibition

**Figure 4:**
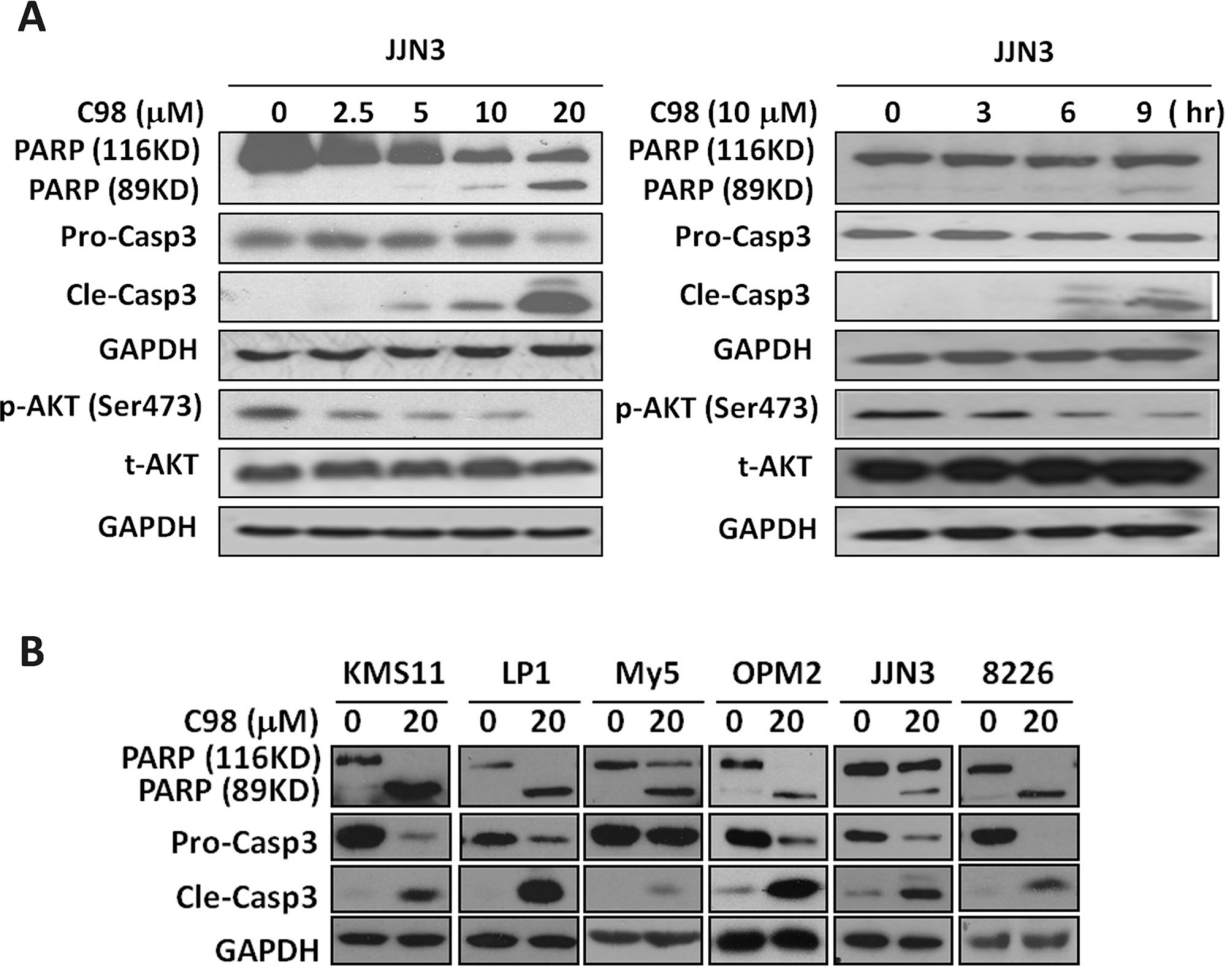
C98 activates apoptotic signaling in MM cells **(A)** JJN3 cells were treated with C98 (0, 2.5, 5, 10, 20 μM) for 24 hr or 10 μM for 0, 3, 6, or 9 hr, followed by immunoblotting for the expression of p-AKT, t-AKT, pro-caspase-3 (Pro-Casp3), and cleaved caspase-3 (Cle-Casp3). **(B)** KMS11, LP1, OCI-My5, OPM2, JJN3, and RPMI-8226 were treated with C98 (20 μM) for 24 hr, followed by immunoblotting for the expression of PARP, Pro-Casp3, and Cle-Casp3. GAPDH was used as a loading control.

**Figure 5:**
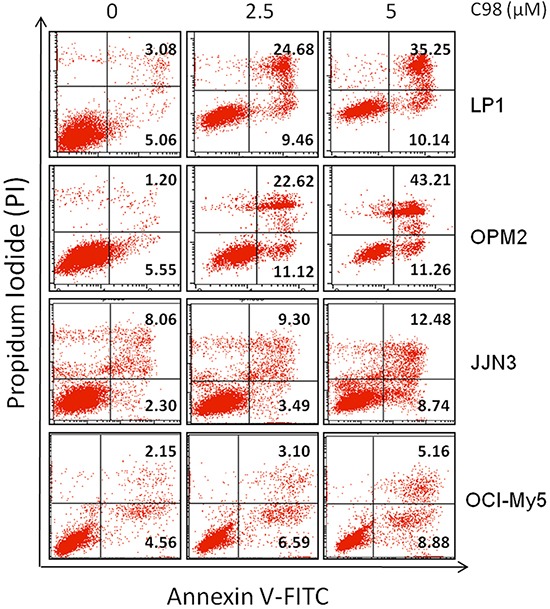
C98 induces apoptosis of MM cells LP1, OPM2, JJN3, and OCI-My5 cells were treated with C98 at 0, 2.5 or 5 μM for 24 hr. Cells were then stained with Annexin V-FITC and propidium iodide (PI), followed by analysis on a flow cytometer. The number in the right quads of each panel means the percentage of Annexin V positive cells.

The above studies have demonstrated that C98 was a specific inhibitor of Class I PI3Ks, to find out whether this compound could induce MM cell apoptosis by targeting PI3K, JJN3 cells were treated with C98 with increased duration or concentrations. As shown in Figure 4A, C98 inhibited AKT phosphorylation and activated caspase-3 in a time- and concentration-dependent manner. To be noted, AKT inhibition occurred earlier or at a lower concentration than caspase-3 cleavage, suggesting that C98-induced MM cell apoptosis was associated with AKT inactivation or PI3K inhibition. To answer whether C98 could induce apoptosis in other MM cells, 5 more MM cells were subject to the study. As shown in Figure 4B, all cell lines examined were triggered to apoptosis because both caspase-3 and PARP were cleaved. Cell apoptosis induced by C98 was further confirmed by the increased fraction of positive cells stained with Annexin V (Figure 5) [27]. To show whether activated PI3K signals can rescue C98 effects on MM cells, both OPM2 and RPMI-8226 cells were treated with IGF-1 alone or together with C98 for 48 hr followed by MTT assay. It showed that IGF-1 could partly abolish C98-decreased MM cell proliferation (Supplementary Figure S3), further convincing C98 targeted the PI3K/AKT signaling pathway.

### C98 is an orally available PI3K inhibitor with robust anti-myeloma activity

**Figure 6:**
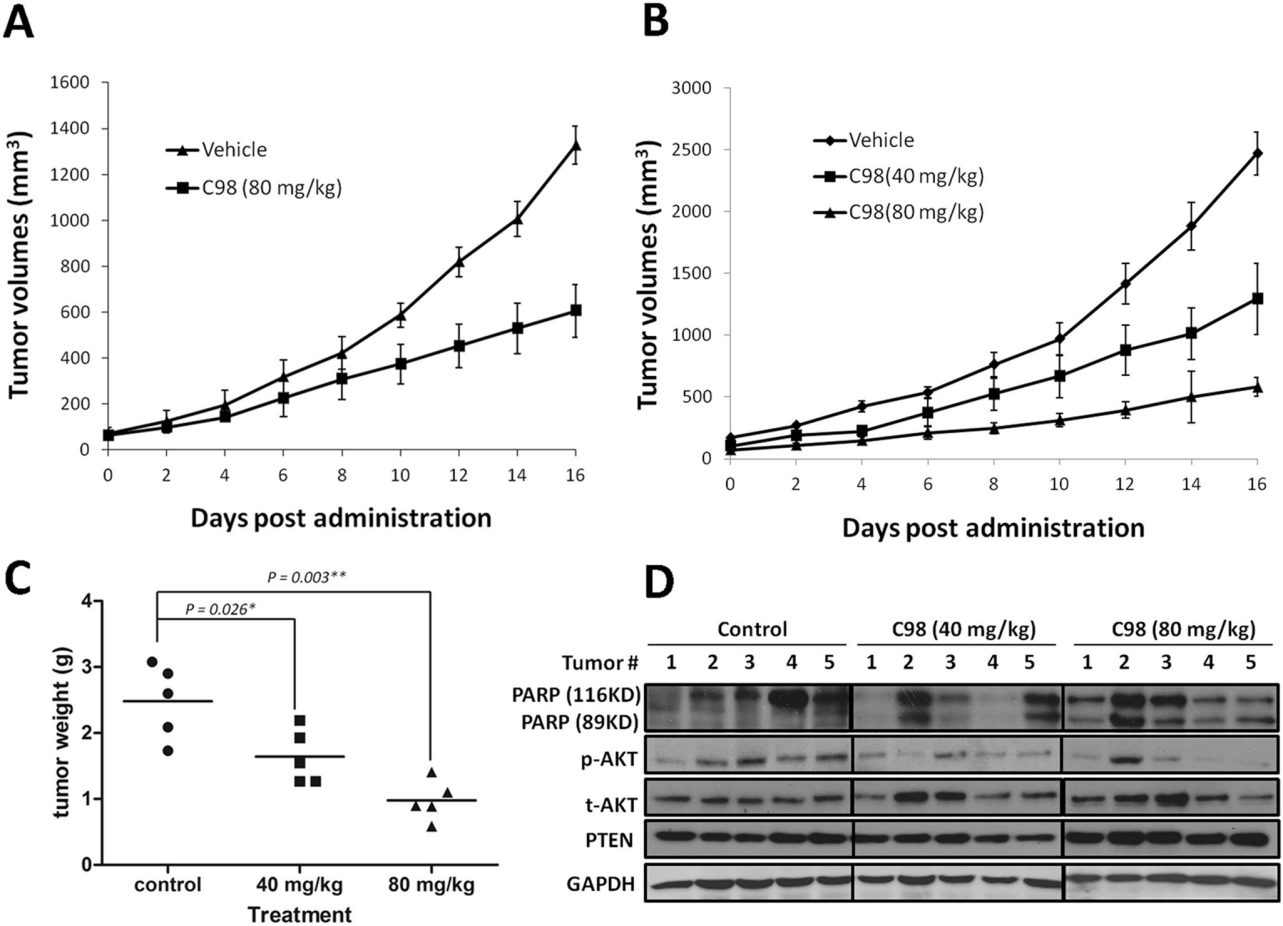
C98 delays myeloma tumor growth in xenograft mice models OPM2 **(A)** and JJN3 **(B)** were injected subcutaneously into nude mice with a density of 10 million cells/site/mouse. When tumors were palpable, mice (*n* = 5/group) were orally given C98 (80 mg/kg to OPM2 mice, 40 and 80 mg/kg to JJN3 mice) in PBS containing 10% Tween 80 and 10% DMSO daily for continuous 16 days. Tumor volumes were monitored every other day. **(C)** Tumor weight from the JJN3 model was measured at the end of the experiment. **(D)** JJN3 cells-derived tumor samples from each group were subject to immunoblotting analysis for the expression of PARP, p-AKT, t-AKT, and PTEN. GAPDH was used as a loading control.

To further evaluate the *in vivo* anti-myeloma activity of C98, two independent MM xenograft models in nude mice were established with two human MM cell lines, OPM2 and JJN3, followed by oral administration of C98. We first evaluated the therapeutic effects of C98 on OPM2 using 80 mg/kg, a dose less than 1/10 of the oral LD_50_ for mice. In the 16-day treatment, tumors were decreased to 45% of the vehicle control (1328.3 ± 82.5 v.s. 605.8 ± 115.7 mm^3^ at the end of the experiment, Figure 6A). This experiment was further confirmed in another xenograft model generated with JJN3, a dexamethasone-resistant MM cell line [28]. In this model, mice were randomly divided into three groups and received vehicle, 40, and 80 mg/kg of C98, respectively. C98 at 80 mg/kg decreased tumor growth to 76.5% (from 2469.4 ± 174.6 mm^3^ decreased to 581.2 ± 73.2 mm^3^) in 16 days (Figure 6B). Notably, C98 at 40 mg/kg also markedly delayed tumor growth (from 2469.4 ± 174.6 mm^3^ decreased to 1293.1 ± 289.7 mm^3^, or 48% decrease) (Figure 6B). The tumor weight was consistent with the dynamic tumor growth analysis (Figure 6C). However, C98 had no effects on body weight of mice in neither models (Supplementary Figure S4). There were no significant changes in blood cell count, platelet count, or hemoglobin measurement in mice treated with C98 (Data not shown).

To evaluate whether C98 delayed MM tumor growth was associated with PI3K inhibition, we measured AKT phosphorylation levels in tumors developed from JJN3 after C98 treatment. As shown in Figure 6D, AKT phosphorylation was decreased by C98 at both 40 and 80 mg/kg doses, while total AKT and PTEN were not markedly affected. Notably, cleavage of PARP was found in the MM tumors excised from C98-treated mice, especially in the high dose group (Figure 6D). We also measured the concentration of C98 in the tumors at the end of the experiment by LC-MS/MS. It showed that the average concentration of C98 in tumor tissues in the 80 mg/kg-treated group was 622.4 ± 374.8 ng/g, which suggested that C98 penetrated into the tumor tissues.

### The pharmacokinetic analysis of C98

To further characterize C98 in the treatment of MM, we determined its pharmacokinetic parameters. One bolus of 40 mg/kg of C98 was orally administrated, blood samples were then collected at the time points of 0.167, 0.5, 1, 2, 4, 6, 8, and 24 hr. LC-MS/MS determined the concentration of C98 in the plasma, and found that C98 was detected in the plasma at 10 min. The median peak concentration (Cmax) was 367.0 ng/mL, the time to maximal concentration (Tmax) was 0.5 hr, the half-life (t_1/2_) was 2.03 hr, and the mean residence time (MRT) was 4.48 hr (Supplementary Figure S5). These data showed that C98 could be rapidly absorbed.

## DISCUSSION

PI3K inhibitors have been identified by several strategies including structure-activity relationship directed design [29], gene-based high throughput screening [15], affinity selection coupled mass spectrometry screening [30], high content screening [31] and virtual screening [32]. Among these strategies, except for VS, all other methods require most advanced techniques and equipments, such as robotic facility, high resolution of fluorescent microscopes, and mass spectrometers, which are very cost and time-consuming. In contrast, by taking advantage of the in-depth understanding of the crystal structure, the molecular mechanism of known PI3K inhibitors, the advancement of molecular docking, and the availability of the large small chemicals libraries, VS is therefore regarded as a reliable, cost-effective and time-saving method to identify PI3K inhibitors [33, 34]. By using the *Glide* HTVS coupled SP-XP mode, C98 was identify as a specific inhibition of PI3K enzymes.

Molecular docking and MD stimulations confirmed that the H-bonds and van de Waals contacts are important for the interference of C98 with the ATP active pocket of PI3Ks. These interactions established between specific groups of C98 and conserved hydrophobic residues of the ATP-binding cleft may account for its potency against PI3K and selectivity profile over protein kinases. C98 interacts with and inhibits PI3Kα, β, δ, and γ isoforms probably because these PI3Ks share conserved domains especially in the active pockets. This is supported by the enzyme reaction assays which showed that C98 inhibits human PI3Kα, β, δ, and γ at nanomolar or low micromolar concentrations but does not suppress PDK1 or mTOR activity. Inhibition of PI3K is also demonstrated by the cell-based assays because C98 treatment entailed a decrease of AKT phosphorylation. Consistent with abrogation of AKT function, C98 decreases phosphorylation of p70S6K and 4E-BP1 proteins, two signature substrates in the PI3K/AKT/mTOR signaling pathway. However, C98 does not show markedly suppression on other associated pathway signals such as IGF-1, ERK, p38, c-Src, and STAT3. Therefore, all these selected inhibition on PI3K suggests C98 is a preferred inhibitor of the PI3K isoforms.

C98 displays robust activity against the PI3K/AKT signals which is critical for cell proliferation and cell survival, therefore C98 treatment led to growth inhibition and apoptosis of multiple MM cell lines. PTEN is a negative modulator of the PI3K signaling, thus the PTEN expression level sometimes plays a key role in the treatment with PI3K inhibitors [35, 36]. In this study, C98 induces apoptosis in both PTEN-deleting and PTEN-expressing cells, suggesting C98 is independent of PTEN expression status. This inhibition was also found in the *in vivo* study because C98 delays MM tumor growth in both PTEN-deleting and PTEN-expressing xenograft models. The phosphorylation level of AKT is correlated with the decrease of tumor volumes and weight. Therefore, the anti-MM activity of C98 is highly associated with its suppression on the PI3K signaling pathway.

The pharmacokinetics analysis on C98 *in vivo* showed C98 is probably rapidly absorbed and distributed because the Tmax is 0.5 hr and the t_1/2_ is around 2 hr. Although we did not measured the concentration in other tissues and organs, C98 was found in the MM tumor tissues with an average concentration up to 622.6 ng/g, suggesting that C98 can penetrate into the tumor tissues where it exerts its anti-myeloma activity. It also suggests C98 has a reliable oral activity. However, to be noted is that C98 is rapidly absorbed and its half-life is relatively short. Therefore, its structure should be optimized to increase its circulation time and to improve its efficacy.

In conclusion, a high throughput VS-based screen identified a novel PI3K-specific inhibitor, which was confirmed by a series of biochemical, cellular and animal studies. The robust oral activity and well tolerance highlights its candidacy for MM treatment.

## MATERIALS AND METHODS

### Antibodies and cell lines

Antibodies against phospho-AKT (Ser473), AKT, phospho-4E-BP1 (S65), 4E-BP1, phospho-mTOR(Ser2448), mTOR, phospho-p70S6K (Ser424), p70S6K, phospho-STAT3 (Thr705), STAT3, phospho-ERK (Thr202/Thr204), ERK, phospho-p38 (Thr180/Thr182), p38, phospho-c-Src (Thr416), c-Src, phospho-IGF-1R (Thr980), IGF-1R, poly (ADP-ribose) polymerase (PARP), and caspase-3 were purchased from Cell Signaling Technologies (Devers, MA). Antibodies against β-actin and GAPDH were obtained from Abgent (Suzhou, China). Horseradish peroxidase-conjugate anti-mouse or anti-rabbit secondary antibodies were purchased from Beyotime (Nantong, China). All MM cell lines and cell culture were described previously [14, 15].

### Structure-based high throughput virtual screening

The high throughput virtual screen was described previously [16]. Briefly, 200,000 compounds from Specs (Delft, The Netherlands) and 600,000 compounds from ChemBridge (San Diego, CA) were docked into the active site of PI3Kγ (3APC) from the RCSB Protein Data Bank [17] and scored using three different scoring modes: high throughput virtual screening (HTVS), standard precision (SP) and extra precision (XP) [16]. Firstly, the top 50% of the structures were saved after the HTVS screening; secondly, these saved structures were redocked and scored by using the *Glide* SP mode, and the resultant top 30% of structures were re-docked and scored by using the *Glide* XP mode. After the XP docking, the top 1,500 compounds ranked by the *Glide* XP scoring after being filtered by the Lipinski's Rule-of-Five employed in MOE were clustered into 150 clusters based on the Tanimoto distance computed from the FCFP_4 fingerprints by using the *Selector* Module in SYBYL 8.1 [18]. From this screen, several potent compounds including C96 [16] and PIK-C98 (C98) (Figure 1A) were identified for further studies.

### Molecular dynamics (MD) simulations and MM/GBSA binding free energy decomposition analysis

MD simulations and MM/GBSA binding free energy decomposition analysis were performed as described previously [19]. Briefly, the *sander* program in AMBER11 were employed to analyze the dynamic interaction patterns between C98 and each Class I PI3K isoform from RCSB Protein Data Bank (PI3Kα: 3ZIM; PI3Kβ: 2Y3A; PI3Kδ: 4AJW; PI3Kγ: 3APC) (http://www.pdb.org/pdb/). Before the MD simulations, the structure of C98 in complex with each PI3K isoform predicted by molecular docking was subjected to minimization. After that, each complex was gradually heated from 0 to 300 K over a period of 50 ps. Then, 5 ns NPT MD simulations were performed with a target temperature of 300 K and a target pressure of 1 atm. All bonds involved in hydrogen atoms were constrained using the SHAKE algorithm, [20] and the time step was set to 2.0 fs. Coordinates were saved every 10 ps for the subsequent analyses. The interactions between each residue in PI3Ks and C98 were characterized using the MM/GBSA free energy decomposition analysis in AMBER11 as reported previously [19, 21].

### Cell viability and apoptosis assays

Cell viability was assessed with the MTT assay as reported previously [22]. To show apoptosis, MM cells were incubated for 24 hr with increasing concentrations of C98 before being stained with Annexin V-FITC and propidium iodide (BioVision Milpitas, CA). Apoptotic cells were measured on a flow cytometer (FACSCalibur^®^, Becton Dickinson) as reported as previously [23].

### Kinase activity in cell-free assay

Kinase activity in the presence of C98 was performed by using the HotSpot technology (Reaction Biology Corp., Malvern, PA, USA) as described previously [15].

### Immunoblotting

The immunoblotting assays were performed as described previously [14]. IRDye 680 goat anti-mouse and IRDye 800CW goat anti-rabbit antibodies were from Odyssey (San Ramon, CA, USA).

### Pharmacokinetic study

Prior to dose administration, nude mice (Shanghai Slac Laboratory Animal Co. Ltd., Shanghai, China) were fasted overnight with free access to water in the regular light-dark cycle. A group of 9 mice received an oral dose of C98 at 40 mg/kg. Blood samples were collected from three mice (*n* = 3) at each time points as follows: 0.167, 0.5, 1, 2, 4, 6, 8, and 24 hr. Blood samples (100 μL) were immediately mixed with 10 μL of EDTA and plasma was obtained by centrifugation at 1.3 × 10^4^ rpm for 10 min. Samples were stored at –80°C for LC-MS/MS analysis. Plasma concentration versus time was analyzed using WinNolin software (version 6.2.1, Pharsight, USA) and the pharmacokinetic parameters maximum drug concentration (Cmax), time to reach maximum drug concentration (Tmax), half life (t_1/2_), area under the plasma level-time curve (AUC) were calculated.

### LC-MS/MS analysis

Concentrations of C98 in plasma and in tumor tissues were analyzed by a LC-MS/MS method as described previously [24]. Chromatographic separation was achieved using an Agela Venusil XBP C18 column (50 × 2.1 mm; 5 mm particle size, Bonna-Agela Technologies, Tianjin, China). The column temperature was maintained at 40°C. Gradient elution at a flow rate of 0.3 mL/min was performed using the mobile phases: A, acetonitrile: water: formic acid (5: 95: 0.1, v: v: v) and B, acetonitrile: water: formic acid (95: 5: 0.1, v: v: v).

The mass spectrometer was operated in the negative ionization mode. The ion transitions for the multiple reaction monitored were: C98, 346.6 (M-H)→312.7; betamethasone (internal standard, IS), 391.1 (M-H)→ 361.4, with a dwell time of 100 ms per transition. After setting the collision energy and mass transition for C98 and the Internal standard, all other parameters were optimized for the best sensitivity. Data was processed by the Analyst 1.5.2 data collection and integration software (AB Sciex, Framingham, MA).

### Myeloma xenograft in nude mice

Totally 50 female SCID in mice from Shanghai Slac Laboratory Animal Co. Ltd were used for the xenograft models with human MM cell lines OPM2 and JJN3, respectively. When tumors were palpable, the OPM2-xenograft mice were randomly divided into two groups. One group as a control was received the vehicle [15], and another group was orally administrated C98 (80 mg/kg) once a day for continuous 16 days. The JJN3 xenografted mice were randomly divided into three groups which were orally administrated vehicle or C98 (40 or 80 mg/kg), respectively. Body weight and tumor volumes were measured every other day as described previously [15]. At the end of this experiment, tumors were collected for further studies. The concentrations of C98 in tumor tissues were also determined by LC-MS/MS as described above. All experiments involved in mice were reviewed and approved by the Review Board of Animal Care and Use of Soochow University.

### Hematology analysis

At the end of the experiment of the *in vivo s*tudies, whole blood samples were collected from the eyes and were immediately subject to complete blood analysis as described previously [14].

### Statistical analysis

For *in vivo* studies, the Mann–Whitney rank sum nonparametric method was used to test for differences between treatment groups in the volumes of the tumors. The student's *t* test was used for comparisons of cell proliferation *in vitro*.

## SUPPLEMENTARY FIGURES


