# Correction: A novel PI3K inhibitor PIK-C98 displays potent preclinical activity against multiple myeloma

**DOI:** 10.18632/oncotarget.27350

**Published:** 2019-12-10

**Authors:** Jingyu Zhu, Man Wang, Yang Yu, Huixin Qi, Kunkun Han, Juan Tang, Zubin Zhang, Yuanying Zeng, Biyin Cao, Chunhua Qiao, Hongjian Zhang, Tingjun Hou, Xinliang Mao

**Affiliations:** ^1^ Jiangsu Key Laboratory of Translational Research and Therapy for Neuro-psycho-diseases, Department of Pharmacology, College of Pharmaceutical Sciences, Soochow University, Suzhou, China; ^2^ Jiangsu Key Laboratory of Preventive and Translational Medicine for Geriatric Diseases, Soochow University, Suzhou, China; ^3^ Department of Medicinal Chemistry, College of Pharmaceutical Sciences, Soochow University, Suzhou, China; ^4^ Department of Pharmaceutical Analysis, College of Pharmaceutical Sciences, Soochow University, Suzhou, China; ^5^ Department of Medicinal Chemistry, School of Pharmaceutical Sciences, Zhejiang University, Hangzhou, China

**This article has been corrected:** Due to errors in the final figure composition, the AKT expression from Figure 2A was accidentally duplicated in Figure 4A. The proper Figure 4A and its updated legend are shown below. The authors declare that these corrections do not change the results or conclusions of this paper.

Original article: Oncotarget. 2015; 6:185–195. 185-195. https://doi.org/10.18632/oncotarget.2688

**Figure 4:**
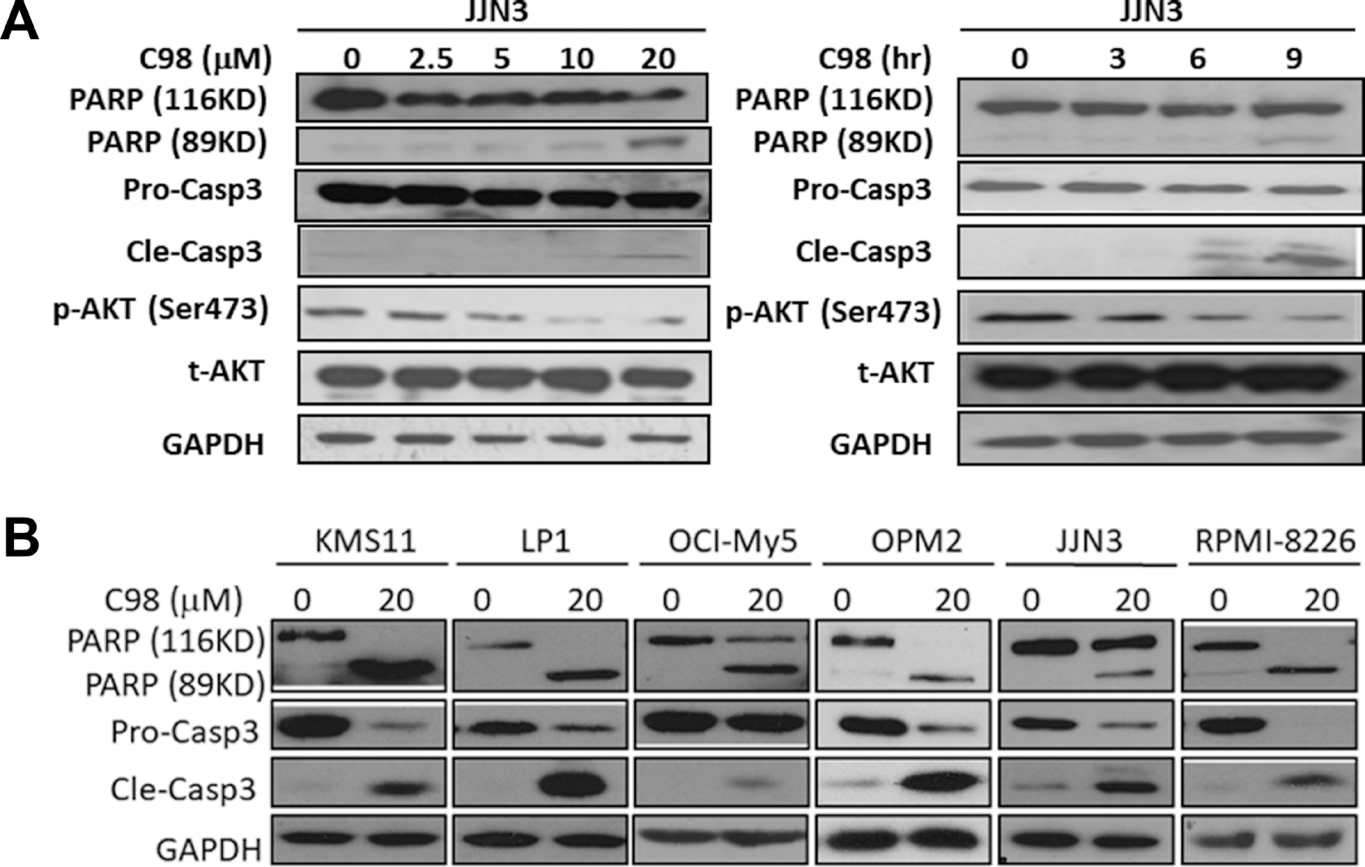
C98 activates apoptotic signaling in MM cells.

(**A**) JJN3 cells were treated with C98 (0, 2.5, 5, 10, 20 μM) for 24 hr or 10 μM for 0, 3, 6, or 9 hr, followed by immunoblotting for the expression of p-AKT, t-AKT, pro-caspase-3 (Pro-Casp3), and cleaved caspase-3 (Cle-Casp3). (**B**) KMS11, LP1, OCI-My5, OPM2, JJN3, and RPMI-8226 were treated with C98 (20 μM) for 24 hr, followed by immunoblotting for the expression of PARP, Pro-Casp3, and Cle-Casp3. GAPDH was used as a loading control.

