# Supplementary material for: A novel PI3K inhibitor PIK-C98 displays potent preclinical activity against multiple myeloma
Source: Oncotarget. 2014 Dec 2;6(1):185–95. doi: 10.18632/oncotarget.2688 (PMC4381587; doi:10.18632/oncotarget.2688)
Supplement: Supplementary file 1 [file oncotarget-06-185-s001.pdf]

SUPPLEMENTARY FIGURES

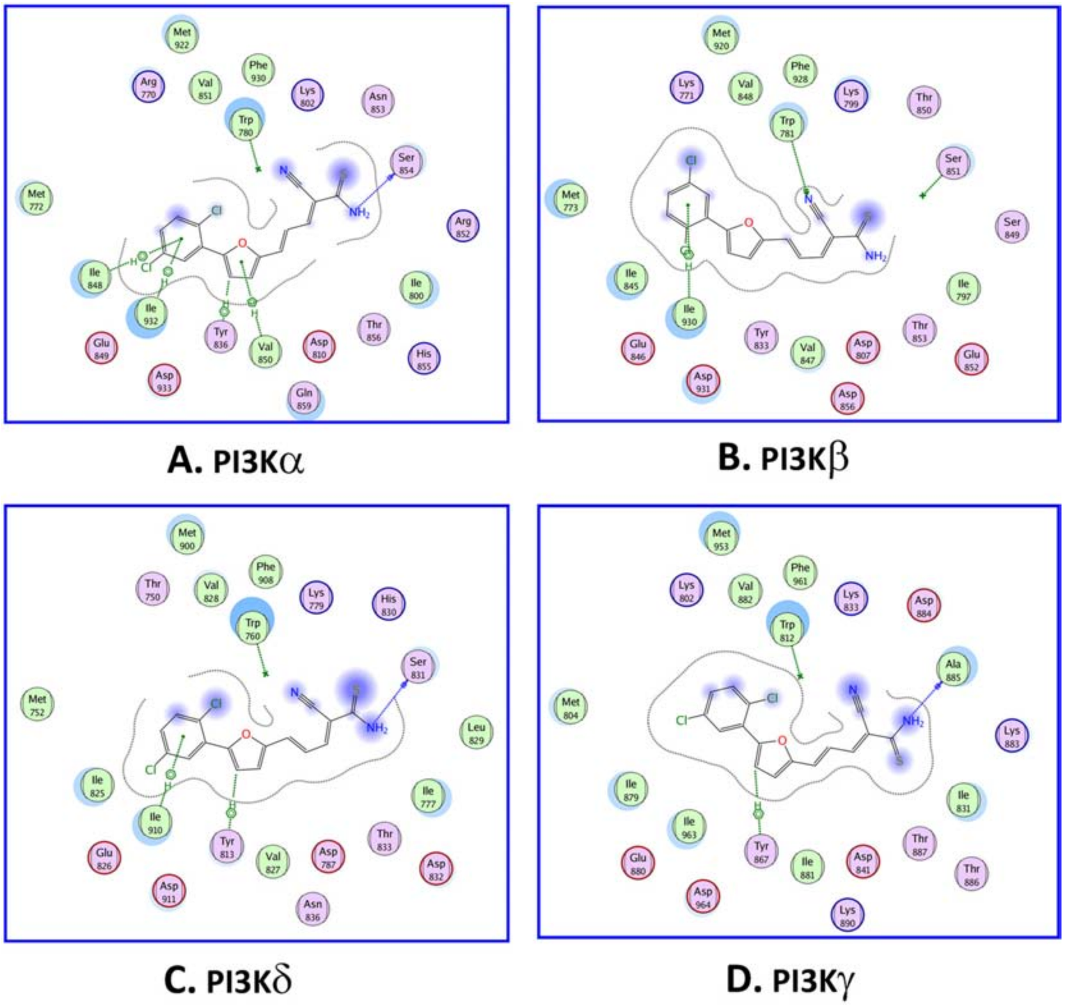

Supplementary Figure S1: The C98-residue interaction spectra for (A) PI3Kα , (B) PI3Kβ, (C) PI3Kδ and (D) PI3Kγ.

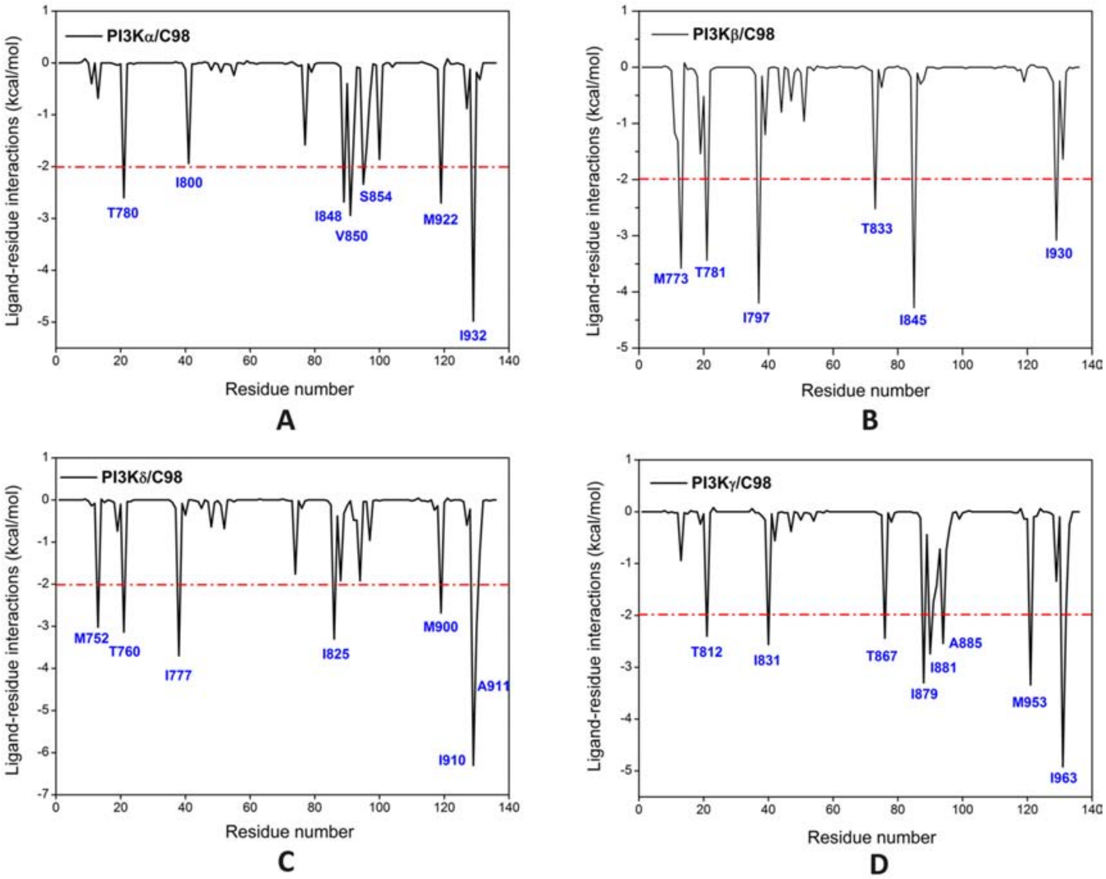

Supplementary Figure S2: MD stimulations of the C98/PI3Ks complexes. 2-D presentations of the interaction patterns between C98 and PI3Kα **(A)** PI3Kβ **(B)** PI3Kδ **(C)** and C98/PI3Kγ **(D)** NH2 in C98 is important for the H-bonds, and the p-chlorophenyl moiety of C98 forms van de Waals contacts with hydrophobic residues.

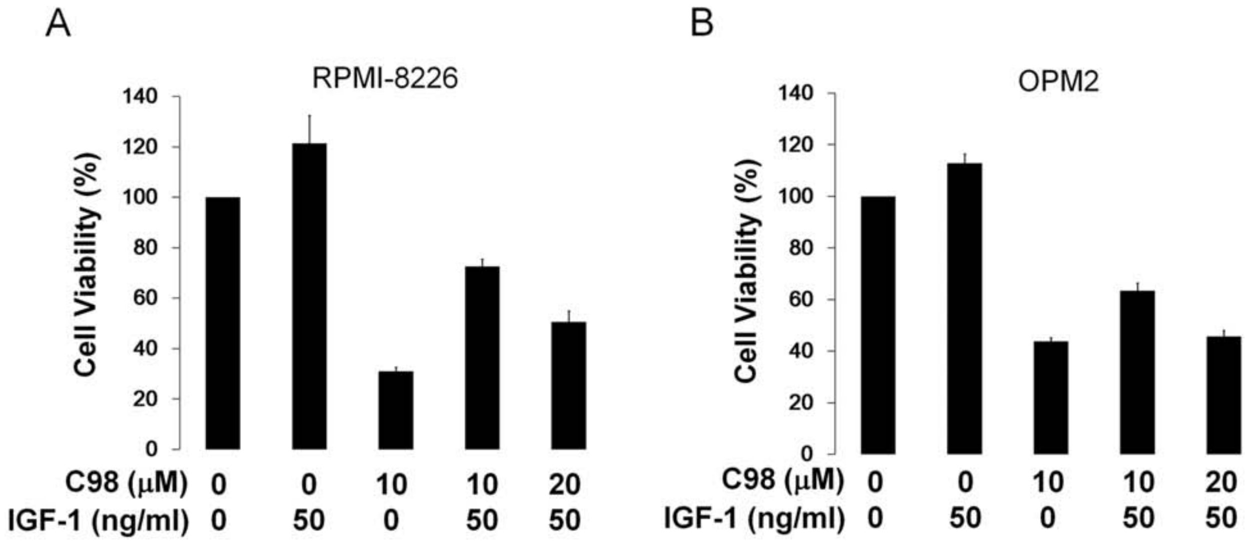

Supplementary Figure S3: IGF-1 rescues C98-decreased MM cell proliferation. OPM2 and RPMI-8226 cells were treated with IGF-1 alone or increased C98 for 48 hr, followed by MTT assay.

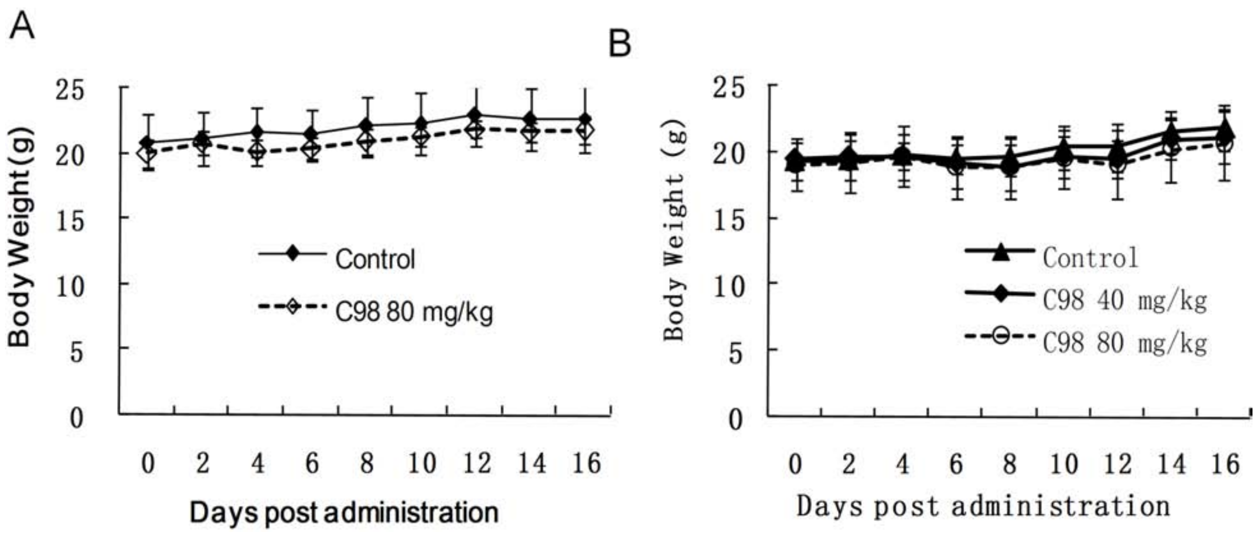

Supplementary Figure S4: C98 does not markedly decrease mice body weight. OPM2 **(A)** and JJN3 **(B)** were injected (s.c.) into nude mice. When tumors were palpable, mice were orally administrated C98 at dosage as indicated for continued 16 days. Mice bodyweight was monitored every other day.

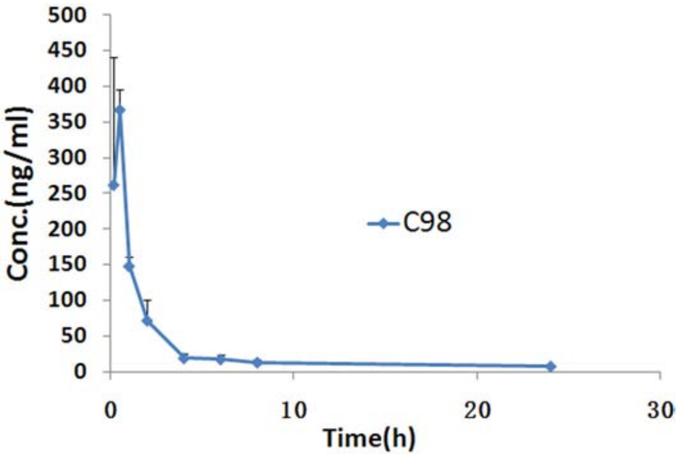

Supplementary Figure S5: The time-concentration profiles of C98 in plasma after oral administration. Nude mice were orally administrated C98 at a dose of 40 mg/kg. Blood species were then collected at a serial of time points (0, 0.167, 0.5, 1, 1.5, 2, 4, 6, 8, 24 hr) and subject to LC-MS/MS analysis after treatment as described in the Methods section. Resultant plasma concentration versus time data was analyzed using WinNolin software.
